# Co‐Development of the CoMUni Intervention: A Social‐Media‐Based Resource to Increase Mental Health Help‐Seeking Behaviours in UK Undergraduates

**DOI:** 10.1111/hex.70400

**Published:** 2025-08-23

**Authors:** N. Wilde, J. Foster, V. Jones, A. Kossivas, Jill Çakmak, O. C. Knight, M. Grzywacz, D. Foxcroft, E. L. Davies

**Affiliations:** ^1^ Oxford Brookes University Faculty of Health and Life Sciences, Psychology Oxford UK

## Abstract

**Introduction:**

Each academic year, numerous students experience mental health problems. Despite this, many avoid seeking any form of help, which can lead to problems worsening. This paper reflects upon the co‐development process of an intervention which aimed to increase help‐seeking behaviours in undergraduate students.

**Methods:**

Online workshops were conducted with a student co‐production team, including the completion of tasks designed to incorporate the eight steps of the Behaviour Change Wheel framework. During the co‐production process, the team made key decisions in relation to the developed intervention's main function, the behaviour change techniques used, and the content presented.

**Results:**

As a result of the process, the team developed CoMUni, a social media‐based intervention sharing other students' experiences of seeking help. After the final workshop, each team member provided feedback through an online form relating to their experiences during the development process. Results of this study illustrate how the values of co‐production were upheld during the development process, with team members feeling actively involved and respected.

**Conclusion:**

This study provides reflective insight into the qualitative approach of co‐production and illustrates its benefits when utilised to develop interventions around university student mental health.

**Participant or Public Contribution:**

A stakeholder advisory group made up of university staff (with experience or interest in student mental health) was consulted during the design of the co‐production workshops. A student team attended co‐production meetings, making key decisions around the functionality and content used in the intervention. Some members of the team also reviewed and commented on the final manuscript.

## 1 Background

Undergraduate students may experience poor mental health due to academic stress, financial worries and establishing new social networks [1]. Previously, 34% of students reported experiencing a mental health problem warranting professional help [2]. Despite the high prevalence of mental health problems, many students are unlikely to seek help when needed, with previous work estimating this to be the case for 60%–80% of students experiencing mental health issues [3].

If students do not seek help when needed, this could lead to further mental deterioration, which could negatively impact many aspects of their lives [2, 4]. Barriers to help‐seeking in the student population include help not being accessible [5], negative attitudes around seeking help and stigma [6], a lack of mental health‐related knowledge [7] and current mental state [8].

**Figure 1:**
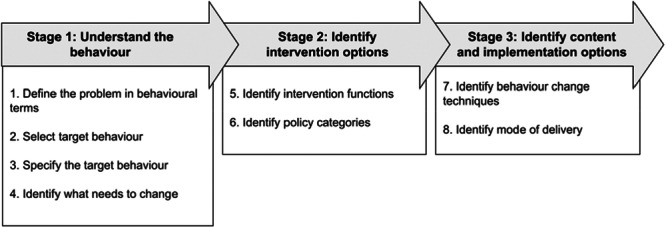
Stages of intervention development, from the Behaviour Change Wheel framework [10].

The present study combined two established approaches, the Behaviour Change Wheel framework (BCW; [9]; Figure 1) and co‐production, to develop a behaviour change intervention which encourages students to seek help for their mental health. The development process utilised the steps and stages of the BCW ([9]; Figure 1), which is a theory‐based approach to intervention design. There is recent evidence that researchers are starting to use the BCW approach to develop interventions aimed at improving mental health‐related behaviours with success [11].

Although a theory‐based approach such as the BCW has several benefits, basing intervention design solely upon theory is not enough to address the complexity of the behavioural problems that an intervention is aiming to change [12]. Allowing stakeholders to contribute is necessary to ensure that an intervention is socially robust and sustainable within its intended context [13, 14]. The development process in this study incorporates a co‐production approach, which aims to create effective interventions while engaging stakeholders as equal partners in the development process [15, 16]. This is achieved by providing support for processes that allow professionals and stakeholders to collaborate, reflecting on each other's unique and valuable insights. For university students, this approach also promotes self‐awareness and peer connection [17, 18].

This combination of using the BCW framework within co‐production approaches has previously been used successfully to develop health behaviour interventions to reduce sedentary behaviour for individuals who had experienced a stroke [19], improve self‐management for those who had experienced a stroke [20], and increase physical activity within families [21]. As far as the authors are aware, this paper presents the first example of working with students to develop a mental health behaviour change intervention using a combination of BCW and co‐production approaches. This study had two main aims: to utilise the BCW framework within a co‐production approach to develop an intervention and to reflect on the feasibility of using a combination approach of the BCW and co‐production in this context.

## 2 Methods

The design of this study included several key stages, which are outlined below.

### 2.1 Preparatory Research

An advisory group meeting was held with five members; all recruited via email. Each had between 2 and 16 years of experience working with university students. While some held roles specifically focused on student mental health, others had a broader interest in supporting student well‐being.

A 1‐h Zoom meeting was conducted on 23 January 2023. From discussions, there were two main takeaways to consider when working with students. Firstly, it is important to be ‘human’; the group advised that sharing any lived experiences might be beneficial to build trust with the student team. The group also advised that during the process, the student team members' views should be affirmed and that they should be allowed to take the lead. This feedback was taken forward to the task design stage.

### 2.2 Workshop Task Design

Workshop tasks were informed by the eight steps of the BCW framework [10] (Figure 1). In some cases, the language was adapted to make it less technical and more easily accessible for the team (e.g., the term ‘intervention’ was replaced by ‘resource’). The aim of each task was to allow the team to draw upon their own experiences as students. Although the tasks were presented and facilitated by the researcher, the co‐production team were kept at the heart of the process and made key decisions about the intervention, including its functionality, content and presentation. An overview of each workshop session, including the tasks set and how these correspond to the BCW, can be found in Table 3.

### 2.3 Recruitment of the Co‐Production Team

The aim was to recruit up to eight co‐production team members, reflecting on the group sizes of previous work [19, 20]. Individuals were able to be co‐production team members if they were current undergraduate students at the host institution. Individuals were recruited using flyers and social media posts. When following the sign‐up link, individuals were presented with a participant information sheet outlining the purpose of the research before being required to provide their consent. Before the initial workshop, each team member was invited to a one‐to‐one meeting with the researcher to ensure they felt as comfortable as possible during the process.

### 2.4 Co‐Production Workshop Process

4 h‐long workshops with the student co‐development team were conducted on Zoom between February and April 2023. These sessions were kept short as previous work has highlighted that many university students struggle when trying to maintain focus during online sessions [22]. Each session began with a short introduction followed by an outline of the session's aims. Tasks were then set to be completed by the team, which included a mixture of individual and team tasks completed through online platforms such as Mentimeter and Google Slides. At the end of each workshop, a recap of the session outcomes was presented, and team members were asked if there was any additional information they would like to add. A full outline of each workshop session and its links to the BCW can be found in Table 1.

**Table 1:** Overview of workshop content.

Session focus and content	Co‐production tasks	Links to BCW stages
Workshop 1	Ice‐breaker: What words would you use to describe life as an undergraduate student?	Stage 1: Understand the behaviour.
Introduction to the researcher and their own lived experience.		
Introduction to the topic of student mental well‐being.	Task 1—Team discussion: ‘What do you think could encourage more students to seek help for their mental wellbeing when they need it?’	Step 4: Identify what needs to change
Outline of research aims.		
Overview of the co‐production process and how sessions would run.		
Workshop 2	Ice‐breaker: What one word sums up last week for you?	Stage 2: Identify intervention options.
Recap of previous workshop.		
Presented with four resources (functions) which aim to increase students' motivation to seek help for their mental well‐being.	Task 2—Feedback task: the team was asked to fill out an online form to answer the following:	Step 5: identify intervention functions
	Do you like this resource?	
	How effective do you think it will be in increasing motivation to seek help?	
	Task 3—Team discussion around the likes, dislikes and concerns of each resource.	
Workshop 3	Task 4—Team discussion task: Rank the eight techniques presented on a scale (dislike—like) and explain reasoning.	Stage 3: identify content and implementation options.
Recap of previous voting outcomes.		Step 7: Identify behavioural change techniques (BCTs)
Overview and explanation of the intervention function chosen to take forward.		
Recap of the term ‘help‐seeking’.		
Presentation of eight BCTs and examples.		
Workshop 4	Task 5—Team discussion:	Stage 3: identify content and implementation options.
Recap of previous workshop.		
Questions and activities relating to the content and presentation of the resource.	What kind of content would you find helpful to see? Is there a particular type of information or content that you would find motivating?	Step 8: Identify mode of delivery
	Do you think it should present a number of student experiences?—If so, how do you think they should differ?	
Providing feedback on the process.	Task 6—Information presentation survey: team members were asked to complete two Mentimeter questions:	
	What platform do you feel would be the most appropriate?	
	How would you like the information to be presented visually?	

### 2.5 Iterative Development Process

The exact direction and content of each task set was determined by the outcomes of previous tasks. Table 2 provides an outline of the outcomes of each task and how these influenced the direction of subsequent tasks.

**Table 2:** List of outcomes of each task and their influence on subsequent tasks.

Task	Outcome/s	Influence on
1	List of aspects that would encourage students to seek help	Deciding appropriate intervention functions to show during Tasks 2 and 3.
		APEASE analysis conducted to select the final intervention function. This function then influenced the BCTs presented during Task 4.
2	Percentage of the team that liked each resource	APEASE analysis conducted to select the final intervention function. This function then influenced the BCTs presented during Task 4.
	Mean level of effectiveness for each resource	
3	List of likes and dislikes of each resource shown	APEASE analysis conducted to select final intervention function. This function then influenced the BCTs presented during Task 4.
4	Ranked order of BCTs	Generation of questions for Task 5.
	Reasoning behind ranking choices	

Following BCW guidelines, the APEASE criteria (Acceptability, Practicability, Effectiveness, Affordability, Side‐effects and Equity) were used at points throughout the process to aid the researcher with judgement making. They were first applied to identify the final function of the intervention, using the outcomes of Tasks 1–3. When calculating an overall APEASE score, affordability, practicability, side effects and equity were scored out of ten by the researcher based upon the content analysis of discussions had by the team during Task 3. Effectiveness and acceptability were provided from the mean effectiveness score and acceptability score, respectively, calculated from data collected during Task 2 (more information about this analysis can be found in the ‘Data Collection and Analysis’ section). Scores of all the APEASE aspects were combined to give each resource a total score out of 60, with a higher score indicating better performance against the APEASE criteria. The APEASE criteria were also referred to by the researcher to aid them in deciding which behaviour change techniques (BCTs) to present during Task 4.

### 2.6 Ethical Considerations

Ethical approval was obtained from the Oxford Brookes University Ethics Committee (UREC Number: 221610).

### 2.7 Data Collection and Analysis

Qualitative data were collected in the form of team discussion transcripts (from Tasks 1, 3, 4 and 5) and text responses to open‐ended questions (Task 2). Transcripts were created by the researcher from the audio recordings of each workshop. All qualitative data collected were analysed using qualitative content analysis, paying attention to themes that illustrated meanings in relation to the questions asked [23].

The procedure for qualitative content analysis included three distinct phases of preparation, organisation and reporting [24]. During the preparation phase, the units of analysis were defined, which in this case were the words and phrases of responses. After this initial preparation, open coding of the responses was conducted, which involved the researcher making notes about the content of the data and organising key phrases and words into relevant categories. Finally, the categories and subcategories that were identified were reported. The analysis was inductive, creating codes from the data instead of going into analysis with a predetermined list [25].

Quantitative data were collected through the completion of online questionnaires during Tasks 2 and 6. During resource feedback (Task 2), data was collected using the Google form platform and included Likert scale data responses to the following two questions: (1) How much do you like the idea (on a five‐point Likert scale from 1 ‘dislike very much’—5 ‘like very much’) and (2) How effective do you feel it would be in encouraging students to seek help? (on a ten‐point scale from 1 ‘Not effective at all’ to 10 ‘Very effective’). Responses to question (1) were analysed to calculate the ‘mean effectiveness score’, which was utilised in the relevant APEASE analysis outlined in the previous section. Responses to question (2) were calculated to produce an ‘acceptability score’ also for use in an APEASE analysis. The acceptability score was calculated by transforming the percentage of the team that liked each resource during Task 2 into a score out of 10 (e.g., if 29% liked the resource, this was a score of 2.9).

Data created during Task 6 was collected using the online platform Mentimeter. During this task, members were asked the following two questions: (1) What platform do you feel would be the most appropriate for presenting this type of information? and (2) How would you like the information to be presented? Members voted for all the options they felt were appropriate and could choose multiple answers. A full outline of the questions and options presented during Task 6 can be found in Supporting materials (Figure S2). The frequency of responses was analysed to highlight perceived appropriateness.

### 2.8 Workshop Evaluation

At the end of the final workshop, team members completed a feedback survey created by the researcher assessing how well the process aligned with co‐production values [26, 27]. They rated eleven statements on five‐point Likert scales (1 = ‘Strongly disagree’ to 5 = ‘Strongly agree’), each reflecting one of five core values outlined by the Stronger Together co‐production toolkit: collaboration, diversity, respect, empowerment and involvement [27] (Table 5). An open‐ended question also invited suggestions for improvement.

The researcher also kept a reflective journal of their experiences throughout the development process, considering both the BCW and co‐production approaches used. In this context, a reflective journal was defined as a ‘personal tale of what went on in the backstage of doing research’ [28] (p. 741). It is important to note that the data collected utilising the reflective journaling method was the researcher's interpretation of events, based on their positionality, a statement of which has been provided (Figure S1 in Supporting Materials). Key insights were summarised to highlight what worked well and considerations for future co‐production with university students.

## 3 Results

### 3.1 Overview of Co‐Production Team and Workshop Attendance

The co‐production team was made up of seven members (aged 20–23), all of whom were current undergraduate students. The majority identified as female (85.7%), heterosexual (71.4%), UK domiciled (71.4%) and in their second year of study (57.1%). Three participants (42.9%) attended all the sessions, two participants (28.6%) attended three sessions and two (28.6%) attended two sessions.

### 3.2 BCW Step 1: Defining the Problem in Behavioural Terms

Previous work conducted by the researcher defined the problem as undergraduate students not carrying out help‐seeking behaviours when experiencing poor mental well‐being [29]. Help‐seeking behaviours are defined as ‘an adaptive coping process that attempts to obtain external assistance to deal with a mental health concern’ [30]. An overview of the problem was presented to the team during the first workshop.

### 3.3 BCW Step 2: Selecting a Target Behaviour

Relevant behaviours were identified through a literature search before the development process. Many behaviours were identified as having an influence on a student's help‐seeking behaviour including avoidance [31], following normative beliefs around students and how they should behave (Velesco et al. 2020), being independent [32], denial [33] and obtaining mental health‐related information [7].

### 3.4 BCW Step 3: Specifying the Target Behaviour

The target behaviour was identified as ‘undergraduate students taking the steps to access a help source when they are experiencing poor mental wellbeing’. This study considered seeking help from both formal (e.g., from a professional) and/or informal (e.g., from family and friends) sources [30]. In line with the BCW framework, a COM‐B analysis of the target behaviour was conducted by the researcher previously and two aspects were identified as being key determinants of help‐seeking behaviours in students: psychological capability and automatic motivation [29].

### 3.5 BCW Step 4: Identifying What Needs to Change

Content analysis of discussions during Task 1 (Workshop 1) identified a number of aspects that the student team felt would encourage them to seek help: less stigma, mental well‐being being discussed positively, providing more information to identify the warning signs of struggling, open discussions of mental well‐being in the university environment, better sign‐posting to helpful resources, making students feel that their issues are valid, providing anonymity, and providing more insight into what the help‐seeking process looks like for students.

### 3.6 BCW Step 5: Identifying Intervention Functions

As a result of an APPEASE analysis conducted by the researcher (full analysis is provided in Table S1 in Supporting Materials), four of the intervention functions fulfilled the criteria: education, persuasion, training and modelling.

These four functions were then taken and developed into resource ideas that were presented to the co‐production team during Task 2 (Workshop 2). The four resources developed were: (1) Help‐seeking information, (2) Positive help‐seeking experiences, (3) Self‐help techniques and (4) A Day in the life representing the functions of education, persuasion, training and modelling, respectively. A full description of each resource and the functions included can be found in Table S2 in Supporting Materials. It's important to note that there was some overlap where resources included multiple functions, but the task presented focused on the most prominent function of the resource. For example, although Resource 2 (positive help‐seeking experiences) was presented as an example of the persuasion function, it was also recognised that there were elements of modelling and education in this resource too. A full outline of the APEASE scores for each resource based on data collected from Tasks 2 and 3 from Workshop 2 can be found in Table 3.

**Table 3:** Overview of APEASE scores.

Resource number and main function	APEASE criteria score					Equity	Total score
	Affordability	Practicability	Effectiveness	Acceptability	Side effects		
1—Education	10	10	4.9	2.9	7	10	44.8
2—Persuasion	10	10	7.3	7.1	10	10	54.4
3—Training	10	8	5.9	8.6	10	10	52.5
4—Modelling	10	8	5.9	5.7	5	10	44.6

The modelling resource ‘A day in the life’ had the lowest APEASE score (44.6). Analysis from Task 3 suggests the team felt this type of resource was not feasible, which had an impact on its practicability score. The team felt that developing an intervention like this would be difficult, especially when seeking out students to create the content, as they felt stigma would put students off doing this:

I'm not sure how the more shy side would feel about it and if you could portray all the different types of students.

Third year student, Female, 23

Discussions also highlighted risks, such as the potential for the students creating content to face negative comments or online trolling, which would be especially harmful for those already in a vulnerable mental state.

The education resource ‘Self‐help techniques’ also scored quite poorly in the analysis (44.8). Although it was perceived as practical by the team, they questioned its effectiveness. Analysis suggested that the team did not feel that just presenting information about help‐seeking was enough, as this is something that they could search for themselves already. The team felt that a resource like this would benefit from something ‘extra’ to make it more relatable or feel more personal to them. There were also concerns raised by the team about the information leading to students incorrectly self‐diagnosing and what the implications of this would be. Due to these concerns, the side‐effect score for this resource was negatively impacted.

The training resource scored highly (52.5) when considering the criteria. This was the resource which scored the highest on acceptability, but discussions made it clear that it very much depended on the type of training that it would be providing.

When you get things like mindfulness or meditation, it is quite an invalidating thing. But the practicality of being trained in a way that you can like, take things on yourself when you notice you're having mental health issues, and you can work it, that would be a lot more practical.

First year student, Female, 21

The team also questioned the practicality of expecting students to have the motivation to engage with a resource like this on top of their already busy learning schedules. However, the team did not identify any negative side effects for this resource.

The persuasion resource scored the highest in this analysis (54.4). Analysis highlighted that the team were aware that a range of student experiences would need to be presented for it to be as beneficial as possible for students, but they could see the potential of a resource like this working effectively. If the help‐seeking experiences presented were real, they felt that this resource could be motivational.

Reflecting on the APEASE scores and the key points made by the team during Workshop 2, it was decided that the function carried forward to Step 7 of the development process would be Persuasion.

### 3.7 BCW Step 6: Identify Policy Categories

Following the NICE (2014) guidance, this intervention aimed to change the help‐seeking behaviours of students on an individual level. Because of this, and in agreement with previous work [19], it was not felt that any of the policy categories were applicable.

### 3.8 BCW Step 7: Identify BCTs

BCTs were considered from the behaviour change taxonomy (BCTTv1 [10]). The 93 techniques were initially filtered down by the researcher, based upon whether they were applicable to the persuasion intervention function. This was achieved by considering guidance of commonly used BCTs for persuasion provided by Michie et al. (2014), but also by the researcher considering what techniques may be beneficial in this context. This identified 17 techniques for consideration in relation to the APEASE criteria (Table S3 in Supporting Materials).

**Figure 2:**
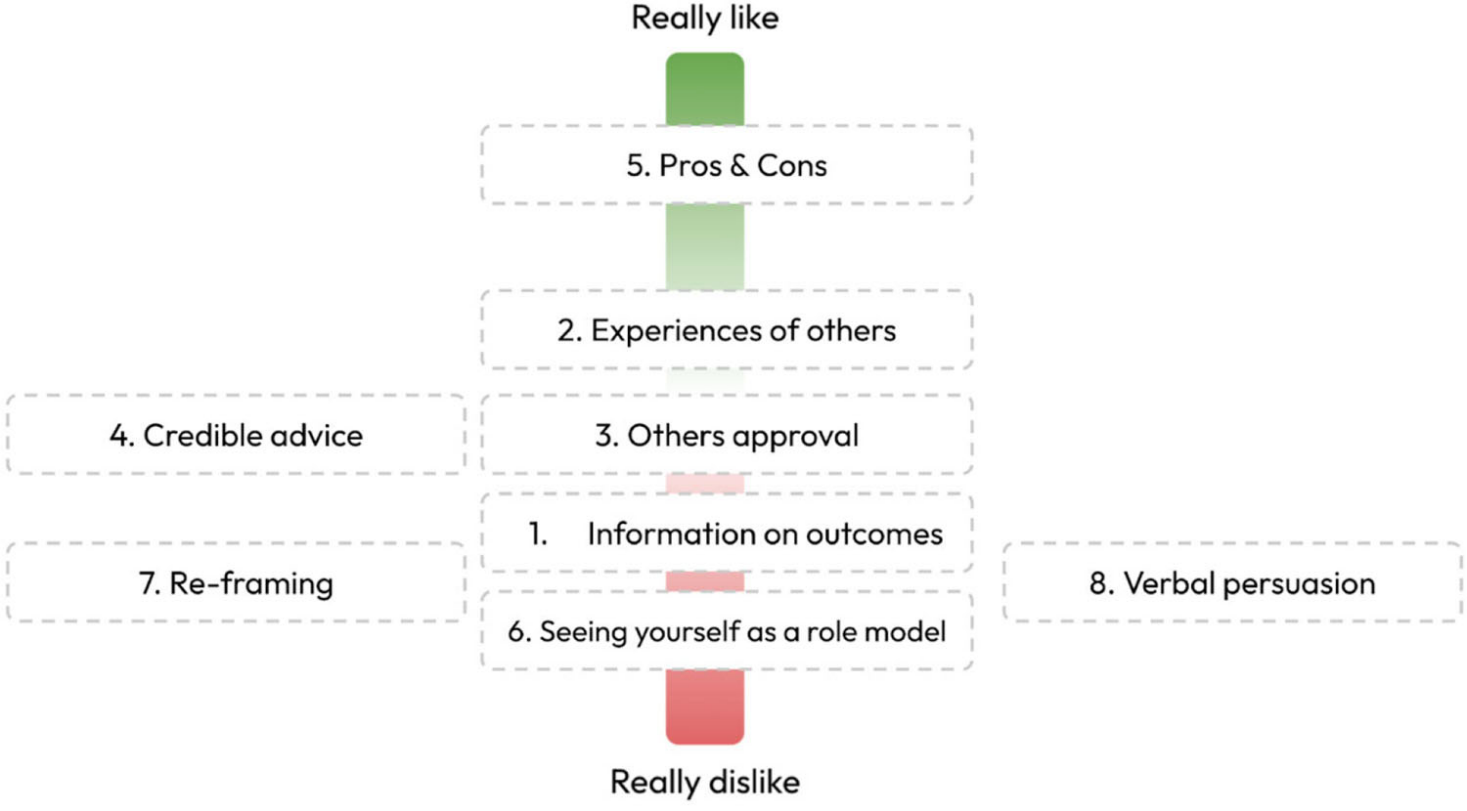
Screenshot of completed Task 4.

The APEASE analysis conducted identified eight suitable techniques, which were presented during Workshop 3 (Table S4 in Supporting Materials). Some of the techniques were re‐named slightly to remove any technical language. Figure 2 presents a screenshot of the completed Task 4, with the team feeling that some of the techniques (e.g., verbal persuasion and others' approval) would not be motivating enough for them to make them want to seek help. They disliked the ‘Seeing yourself as a role model’ technique the most, as they felt it was putting a lot of pressure on their shoulders which they felt uncomfortable with. The team felt that techniques such as ‘information on outcomes’, ‘credible advice’ and ‘reframing’ currently existed on the internet and that they could find this type of information already if they wanted to and the generic and impersonal nature of these techniques was not motivational.

The results of Task 4 highlight the ‘Pros and Cons’ technique as the most liked by the team. During the task, the team explained how they liked the idea of being able to visualise help‐seeking in this way and even if there were cons, they would find this helpful information to have. The team also liked the ‘experience of others’ technique and highlighted that, ideally, they would like to see a combination of the two techniques:

Some of the pros could be outlined and might be others positive experiences. And, like one of the few cons, might be, you're afraid of stigma, or you're afraid that, let's say, therapy might not go well, but when you kind of evaluate it in the context of others positive experiences, it would kind of give you a bit of backing for your own lists.

Third year student, Male, 21

The team liked the idea of hearing about others' positive help‐seeking experiences alongside any challenges they faced during the process. It was decided by the team during Workshop 3 that the intervention should bring together a mixture of two techniques outlined in the BCTTv1: Social Comparison (6.2) and Pros and Cons (9.2).

### 3.9 Step 8: Identify Mode of Delivery

During Task 5 (Workshop 4), the team explained that they wanted the content of the intervention to include information on two main aspects: the help‐seeking process itself and the students' thoughts and feelings. The team wanted information on each student's initial steps to seeking help, any instigating moments, any obstacles they faced, how long the process took, and how the help addressed each student's problems. But the team also felt that the content should describe how each student felt before, during and after the process of seeking help.

It was important to the team that the experiences presented were genuine, outlining the positives but also the challenges the student faced. The team liked showcasing diverse student experiences, allowing them to focus on stories they related to. Some felt they would relate to demographic details, whereas others would relate more to students' experiences and emotions.

During Task 6 (Workshop 4), when asked to indicate platforms they felt would be appropriate for the resource, having a dedicated website and using Instagram had the highest number of votes (Table 4). Discussions highlighted that the team felt that Instagram could be used effectively, especially if further information could be accessed through a link to a dedicated webpage. Results did not highlight any clear preference for how the information should be presented (Table 4), with team members explaining how the content of the intervention was more important than its presentation.

**Table 4:** Task 6 results indicating platform and visual preferences.

	Responses	
	*n*	%
Q1. What platform do you feel would be the most appropriate?		
Dedicated website	4	57%
Through email	1	14%
Instagram	4	57%
Snapchat	1	14%
TikTok	3	43%
YouTube	3	43%
Q2. How would you like the information to be presented?		
Videos using actors	3	43%
Written text	3	43%
Animations	4	57%
Sound/voice only	4	57%
Illustrations/pictures	4	57%

### 3.10 Post‐Session Intervention Development

**Figure 3:**
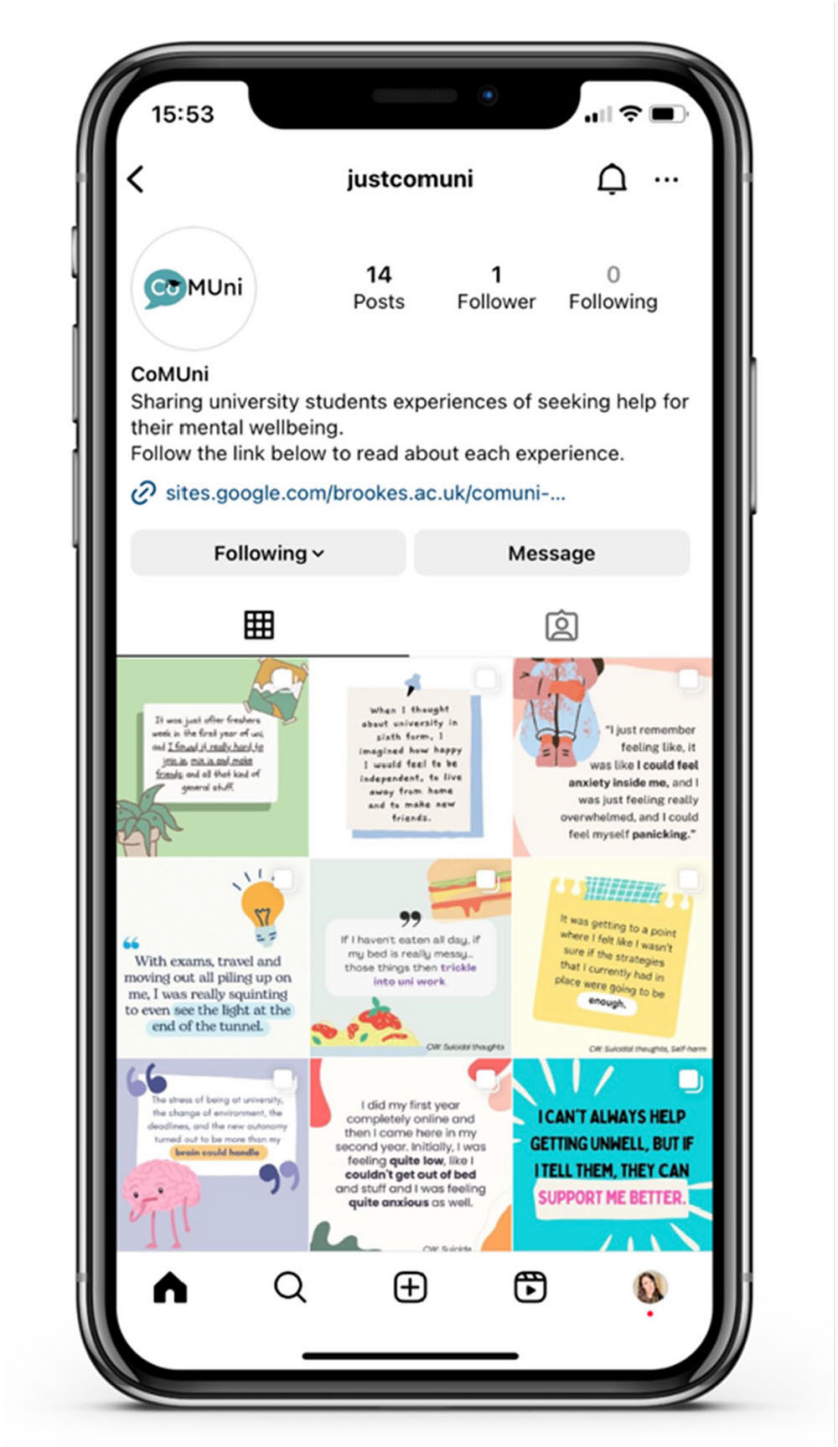
The CoMUni intervention.

After Workshop 4, all the team's decisions were combined to create the final intervention. Discussions had during the tasks highlighted that levels of perceived similarity to the student may affect how engaging they found the information. Considering this, it was decided that only information relating to the actual help‐seeking experience would be provided and that the content would be presented using written text and illustrations. The developed intervention, called CoMUni (**Co**mmunicating **M**ental wellbeing at **Uni**versity, Figure 3), shares a variety of positive help‐seeking experiences provided by undergraduate students.

**Figure 4:**
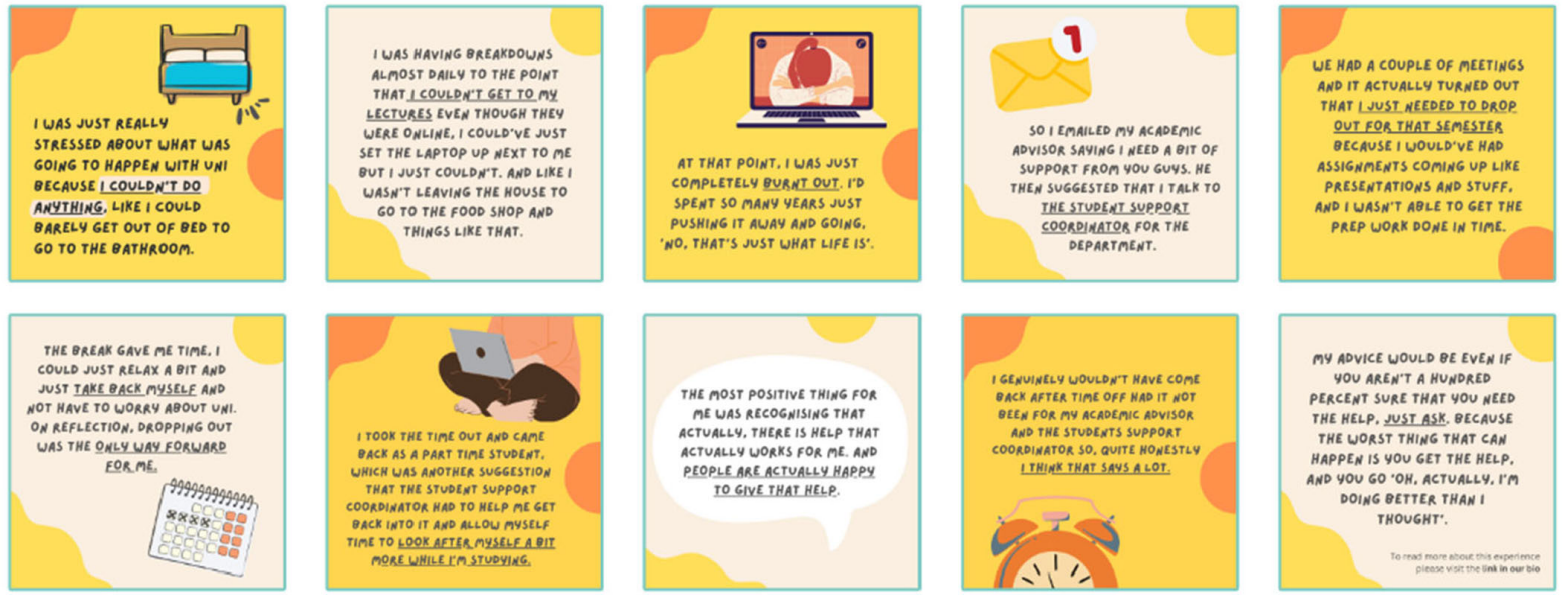
Example of a post with 10 slides outlining a student's help‐seeking experience.

The resource was based primarily on Instagram, with access to a dedicated website containing further information [34]. Each student's experience was presented within a 10‐slide post (Figure 4). These slides contained illustrations and written text to accurately portray the key aspects of their experience, outlining the help‐seeking process itself whilst describing the students' feelings at each stage. Each experience was collected by the researcher through interviewing students who were willing to share their stories anonymously.

### 3.11 Feedback on the Co‐Production Process

Overall, most team members agreed that the co‐production process allowed for effective collaboration, diversity, respect, empowerment and involvement (see Table 5). When asked what they felt would improve the process, some suggested that the team may have felt better connected if it were mandatory to have webcams on. The less confident members of the team stated that they may have put their cameras on if others had done the same.

**Table 5:** Overview of responses from the feedback form.

Co‐production value	Corresponding statement	Mean score (1–5)	Percentage response (*n*)		
			Agree	Neither	Disagree
Collaboration	The team worked well together during the process.	3.9	57.1 (4)	42.9 (3)	0 (0)
	The goals/aims of each meeting were clearly defined and explained.	4.7	100.0 (7)	0 (0)	0 (0)
Diversity	My lived experience and perspectives were valued during the process.	4.6	100.0 (7)	0 (0)	0 (0)
	I felt that I was able to participate fully in the sessions.	4.6	100.0 (7)	0 (0)	0 (0)
Respect	The meetings felt like safe, inclusive and accessible spaces.	4.3	85.7 (6)	14.2 (1)	0 (0)
	I felt like I was respected as a team member.	4.6	85.7 (6)	14.2 (1)	0 (0)
Empowerment	I felt like an equal member of the team.	4.6	100.0 (7)	0 (0)	0 (0)
	I feel that being a part of this process has been beneficial for me.	4.3	85.7 (6)	14.2 (1)	0 (0)
	I feel that this process has allowed me to develop my own skills (e.g., communication, team working or critical thinking skills).	4.0	71.4 (5)	28.6 (2)	0 (0)
Involvement	I felt actively involved in the resource development process.	4.3	71.4 (5)	28.6 (2)	0 (0)
	I feel that I had an influence on the design of the final resource.	4.1	85.7 (6)	14.2 (1)	0 (0)

Reflective journal data highlighted several key points on what worked well and what could be improved in future work. The use of the ice‐breaker questions at the beginning of workshop sessions worked well to encourage team members to speak and build relationships with each other. The researcher sharing their lived experience of mental illness as a student themselves helped to ‘open up the space’ and ensure that the team members felt more comfortable talking about mental health during the process.

Forming relationships between the team members was found to be challenging. The researcher tried to encourage team members to put their cameras on to help with this, but they did not want to make cameras mandatory at the expense of making members feel uncomfortable. In addition, some members of the team were found to contribute more to the team discussions. In the future, it is important to support all team members to contribute as much as they would like to, especially if the team members may experience anxiety with regard to social situations.

## 4 Discussion

This paper describes and reflects on the development process of an intervention which aimed to increase mental health help‐seeking behaviours in undergraduate students. The team created an intervention alongside providing feedback on the development process itself which incorporated the BCW framework within co‐production approaches.

### 4.1 Use of the BCW Framework

Applying the BCW framework was effective and incorporated the context of the co‐production process with ease. The framework provided valuable guidance around how to structure the development process and kick‐started creativity; it provided a starting point for discussions, which was felt to encourage idea generation more so than if the team had been presented with a blank page. For example, in Task 2, it was felt that providing resource examples as opposed to asking the team to create their own resources led to insightful discussions without the team feeling overwhelmed. This contrasts with previous research that has suggested the BCW stunts creativity [19, 20].

One challenge, as was found with previous work [20], was that Stages 2 and 3 of the BCW incorporate many functions and techniques, which require specific technical language to fully understand. A related challenge was how to effectively reduce aspects of the framework so that key decisions could be made by the team during the hour‐long sessions (e.g., it was not feasible to present all 8 functions and 93 techniques). Although the APEASE criteria were helpful in this case to reduce the number of functions and techniques to present during sessions, it could be argued that this took some of the decision‐making away from the team.

### 4.2 Incorporation of Co‐Production Approach and Values

When applied to the context of this study, the co‐production values outlined in the toolkit produced by Stronger Together [27] translated well. The team highlighted that they felt valued, respected throughout the process, actively involved, and liked that they had an influence during the process.

Interactions with the co‐production team were rewarding for the researcher, bringing fresh energy to the development process. Students also found the process beneficial, with feedback indicating it was empowering and helped them to build skills in a safe space, which has been highlighted as especially important in contexts such as mental health where stigma exists [35].

A key challenge in co‐production is balancing power among participants [15, 20]. To address this, the researcher shared their lived experience and clarified expectations in the first workshop, which appeared effective. The most challenging aspect of the process was the amount of time needed. As found in previous work [20], a significant amount of time was spent building relationships with team members and generating the content for each session.

The online nature of the process presented additional challenges which previous face‐to‐face work may not have encountered [19, 20]. Although researcher–team relationships were strong, building connections between team members was more difficult, as reflected in feedback. Future work should include strategies to encourage team building before co‐development begins.

### 4.3 Strengths and Limitations

This study presents an in‐depth and transparent outline of the methods used to develop an intervention to encourage student help‐seeking behaviours, allowing the process to be replicated with ease in the future. The process presented builds on previous work that has combined the BCW framework with co‐production approaches [19, 20, 21], providing insight into its feasibility within a new context. This study also addresses the calls from previous researchers to produce advice and guidance on how to engage students within a co‐production process (Piper and Emmanuel 2019).

A limitation of this study is that the co‐production team was not truly representative of the current student population. This could have been due to students only being recruited from the host university. Encouraging students from multiple universities in the United Kingdom to be part of the co‐development team may lead to a more representative sample. Future work is required on how to engage individuals from a wide range of backgrounds to take part in co‐production processes, a challenge which was also found in previous work [19]. Ensuring that all students are represented in the co‐development process is important to ensure that the developed intervention addresses the needs of all users [36]. The current stigma surrounding mental health within university settings may make engagement challenging [37], so further work is needed to make co‐production processes more accessible.

## 5 Conclusion

This paper outlines and reflects upon an intervention development process which incorporates the BCW framework alongside co‐production values. Although there were some challenges, the use of this approach in this context had many benefits and led to the development of an intervention which uses mechanisms rooted in established behavioural theory. This study provides a rigorous account of how to utilise a combination of BCW and co‐production approaches with a student team to develop an intervention, knowledge which may be useful for future researchers to consider.

## Author Contribution

N.W., E.L.D and D.F. contributed to the design and implementation of the research (including the intervention development process). N.W facilitated the workshops with the student co‐production team, which included J.F, V.J, A.K, J.C, O.C.K., and M.G. All authors worked on the preparation of the manuscript.

## Conflicts of Interest

The authors declare no conflicts of interest.

## Supporting information

Figure S1. Researcher positionality statement. Figure S2. Overview of questions presented during Task 6. Table S1. Identification of intervention functions to present to co‐production team. Table S2. Overview of resources presented during task two. Table S3. Identifying appropriate BCTs against the APEASE criteria. Table S4. Overview of behaviour change techniques presented.

## Data Availability

The data that support the findings of this study are available from the corresponding author upon reasonable request.
