# Supplementary material for: Co‐Development of the CoMUni Intervention: A Social‐Media‐Based Resource to Increase Mental Health Help‐Seeking Behaviours in UK Undergraduates
Source: Health Expect. 2025 Aug 23;28(4):e70400. doi: 10.1111/hex.70400 (PMC12374232; doi:10.1111/hex.70400)
Supplement: Supplementary file 1 — Figure S1. Researcher positionality statement. Figure S2. Overview of questions presented during Task 6. Table S1. Identification of intervention functions to present to co‐production team. Table S2. Overview of resources presented during task two. Table S3. Identifying appropriate BCTs against the APEASE criteria. Table S4. Overview of behaviour change techniques presented. [file HEX-28-e70400-s001.docx]

# Supplementary Materials

|  | ‘The researcher conducting the research was a 33-year-old White British Heterosexual female, using She/her pronouns. She was born in the UK and grew up within a working-class family living in the West Midlands. She has experienced generalised anxiety disorder since childhood and was officially diagnosed at 23. She was the first generation of her family to go to university and was educated to PhD level. During her undergraduate studies, she experienced mental health issues but did not seek help for many years which led to things getting progressively worse. She is interested in learning more about university student mental health and help-seeking behaviours as she recognises the challenges and wants to help future students.’ |  |
| --- | --- | --- |

Figure S1. Researcher positionality statement

| What platform do you feel would be the most appropriate for presenting this type of information? (Please select all that are appropriate)   - Dedicated Website - Email - Instagram - Snapchat - TikTok - Youtube   How would you like the information to be presented? (Please select all that are appropriate)   - Video - using actors - Video - Animations with voice over - Illustrations - Sound/ voice only (e.g. Podcast) - Written text |
| --- |

Figure S2. Overview of questions presented during Task 6

Table S1. Identification of intervention functions to present to co-production team

| Intervention function  Definition | Meets APEASE? | Rationale |
| --- | --- | --- |
| Education  Increasing knowledge or understanding | YES | Affordability: Could be implemented within a realistic research budget.  Practicability: Information around mental wellbeing and help-seeking could be provided for students.  Effectiveness: Previous work has suggested that increasing mental health knowledge can lead to increases in help-seeking behaviours (Smith & Socket, 2011)  Acceptability: Previous work has suggested that increasing knowledge is acceptable, but this will be fully determined during the co-production meeting by the team.  Side-effects: Potential for information overload - students already have a lot of information presented to them.  Equity: For individuals with learning disabilities, they may find this function as effective as others. |
| Persuasion  Using communication to induce positive feelings or stimulate action | YES | Affordability: Could be implemented within a realistic research budget.  Practicability: Stories of other students’ positive help-seeking experiences could be implemented.  Effectiveness: Uncertain about this specific context, but persuasion has worked effectively at changing other health related behaviours such as increasing physical activity (Aloulou et al., 2023).  Acceptability: Previous work has suggested that persuasion could be acceptable, but this will be fully determined during the co-production meeting by the team.  Side-effects: Not all students may respond to persuasion, it could make some feel worse.  Equity: None identified. |
| Incentivisation  Creating an expectation of reward | NO | Creating an expectation of reward was not felt to be an appropriate or sustainable function to use to encourage student help-seeking behaviours in the long term. |
| Coercion  Creating an expectation of punishment or cost | NO | Creating an expectation of punishment for not seeking help was not felt to be an appropriate function to use within the context of mental wellbeing. |
| Training  Imparting skills | YES | Affordability: Could be implemented within a realistic research budget.  Practicability: Students could be provided with self-help skills based on mental health first aid.  Effectiveness: Previous work has found imparting skills in this context (such as mental health first aid) to be beneficial in reducing help-seeking barriers such as negative attitudes (Hadlaczky et al., 2014)  Acceptability: Previous work has suggested that increasing skills is acceptable, but this will be fully determined during the co-production meeting by the team.  Side-effects: Potential for anxiety around learning new skills, some individuals may pick the skills up easier than others.  Equity: For individuals with learning disabilities, they may not find this function as effective as others. |
| Restriction  Using rules to increase target behaviour by reducing opportunity to engage in competing behaviours | NO | Enforcing rules to increase help-seeking behaviour was not felt to be appropriate or practical within this research context. |
| Environmental restructuring  Changing the physical or social context | NO | Changing the physical or social context did not feel practical or obtainable within this context. |
| Modelling  Providing an example for people to aspire to or imitate | YES | Affordability: Could be implemented within a realistic research budget.  Practicability: Student role models who have successfully completed the help-seeking process could be presented  Effectiveness: Uncertain about this specific context, but modelling has worked effectively at changing other health related behaviours such as increasing fruit and vegetable consumption (Lowe et al., 2004) and illness self-management (Hutchings et al., 2022)  Acceptability: Previous work has suggested that modelling is acceptable, but this will be fully determined during the co-production meeting by the team.  Side-effects: Encouraging comparison may result in some individuals feeling worse.  Equity: None identified. |
| Enablement  Reducing barriers to increase capability or opportunity | NO | Reducing barriers to increase capability or opportunity did not feel practical or realistic within this context. |

Table S2. Overview of resources presented during task two

| Resource | | Description | Main function  (other functions) |
| --- | --- | --- | --- |
| 1 | Help-seeking information | Presents information to undergraduate students relating to seeking help for poor mental wellbeing. Information would include:  Methods of seeking help available for undergraduate students.  Explanation of the exact process and steps of the methods outlined.  Why it’s important to seek help as a student. | Education (Training) |
| 2 | Positive help-seeking experiences | Presents examples of positive experiences of seeking help from other university students.  These examples would include information on carrying out the help-seeking process itself and what happens to the individual after seeking help for their mental wellbeing. | Persuasion  (Education, Modelling) |
| 3 | Self-help techniques | Trains students in a variety of self-help methods that can be used when experiencing poor mental wellbeing.  Examples of self-help techniques that could be included could be basic elements of MHFA or CBT. | Training (Education) |
| 4 | A day in the life | Presents ‘day in the life’ experiences of undergraduate students from a variety of different backgrounds.  These stories will have a main focus on their general wellbeing, exploring the challenges they face as a student with regards to this and will be presented by the students themselves. | Modelling  (Education) |

Table S3. Identifying appropriate BCTs against the APEASE criteria

| Related BCTs | | Meets the APEASE criteria in the context of help-seeking? |
| --- | --- | --- |
| 2.2 | Feedback on behaviour | No - Providing feedback on help-seeking behaviours may not be beneficial for everyone and may negatively impact mental health. |
| 2.6 | Biofeedback | No - Not suitable for use within this context. |
| 2.7 | Feedback on outcome(s) of behaviour | No - Not practical for everyone as they may not have previous experience to consider. |
| 4.3 | Re-attribution | No - Discussions around help-seeking behaviours and reasons behind these may be triggering or upsetting for some people. |
| 5.1 | Information about health consequences | Yes |
| 5.2 | Salience of consequences | No - Would not be effective in this context, could be upsetting and not helpful. |
| 5.3 | Information about social and environmental consequences | No - Consequences may not be the same for everyone. |
| 5.6 | Information about emotional consequences | No - Emotional consequences may not be the same for everyone - positive outcomes can’t be guaranteed so could be misleading |
| 6.2 | Social comparison | Yes |
| 6.3 | Information about others’ approval | Yes |
| 9.1 | Credible source | Yes |
| 9.2 | Pros and cons | Yes |
| 13.1 | Identification of self as role model | Yes |
| 13.2 | Framing/ reframing | Yes |
| 13.5 | Identity associated with changed behaviour | No - Not appropriate in the context of seeking help. |
| 15.1 | Verbal persuasion about capability | Yes |
| 15.3 | Focus on past success | No - Not practical as some individuals may not have any previous experiences to reflect upon |

 Table S4 Overview of behaviour change techniques presented

| BCTTv1 item | Overview of technique presented in session |
| --- | --- |
| 5.1 - Information about health consequences | 1. Information on outcomes  Providing information about the positive outcomes of seeking help for your mental wellbeing:  Outlining the positive effects or changes that may occur.  Explaining how outcomes may positively influence academic/social aspects and physical health. |
| 6.2 - Social comparison | 2. Experiences of others  Providing information about others positive experiences of seeking help.  Providing an outline of their journey  Presenting overview of their feelings before, during and after seeking help. |
| 6.3 - Information about others approval | 3. Others approval  Presenting survey results that highlight as a population, students approve of seeking help for your mental wellbeing. |
| 9.1 - Credible source | 4. Credible advice  Presenting a speech from a wellbeing professional to emphasise the importance and benefits of students seeking help at the right time. |
| 9.2 - Pros and cons | 5. Pros and Cons  Prompt questions to get you to think about and weigh up the advantages and disadvantages of seeking help at the current time. |
| 13.1 - Identification of self as a role model | 6. Seeing yourself as a role model  Presenting information that explains how, by seeking help for your own mental wellbeing, you may be helping to address the current stigma. This could make your friends feel more willing to seek help for themselves. |
| 13.2 - Framing/ reframing | 7. Re-framing  Consider help seeking in more positive terms - refilling your cup, recharging your batteries etc. |
| 15.1 - Verbal persuasion about capability | 8. Verbal persuasion  Providing information telling you that you can successfully seek help, despite the barriers you may currently be facing. |
